# *Antrodia cinnamomea* polysaccharide improves liver antioxidant, anti-inflammatory capacity, and cecal flora structure of slow-growing broiler breeds challenged with lipopolysaccharide

**DOI:** 10.3389/fvets.2022.994782

**Published:** 2022-10-10

**Authors:** Jinling Ye, Chang Zhang, Qiuli Fan, Xiajing Lin, Yibing Wang, Mahmoud Azzam, Rashed Alhotan, Abdulmohsen Alqhtani, Shouqun Jiang

**Affiliations:** ^1^State Key Laboratory of Livestock and Poultry Breeding, Key Laboratory of Animal Nutrition and Feed Science in South China, Guangdong Provincial Key Laboratory of Animal Breeding and Nutrition, Ministry of Agriculture and Rural Affairs, Institute of Animal Science, Guangdong Academy of Agricultural Sciences, Guangzhou, China; ^2^Department of Animal Production College of Food and Agriculture Sciences, King Saud University, Riyadh, Saudi Arabia

**Keywords:** *Antrodia cinnamomea* polysaccharide, slow-growing broiler, LPS, liver, cecal microflora

## Abstract

Lipopolysaccharides (LPS) induces liver inflammatory response by activating the TLR4/NF-κB signaling pathway. *Antrodia cinnamomea* polysaccharide (ACP) is a medicinal mushroom that can protect from intoxication, liver injury, and inflammation. Nevertheless, the effect of ACP on the liver antioxidant, anti-inflammatory capacity and cecal flora structure of LPS-challenged broilers remains unclear. The aim of this experiment was to investigate the effects of ACP on the anti-oxidative and anti-inflammatory capacities of the liver, and cecal microbiota in slow-growing broilers stimulated by LPS. A total of 750 slow-growing broilers (9-day-old) were assigned to five treatments with 6 replicates of 25 chicks per replicate: a control diet, the chicks were fed a control diet and challenged with LPS. Dietary treatments 3 to 5 were the control diet supplemented with 100, 200, 400 mg/kg ACP challenged with LPS, respectively. The groups of 100 mg/kg ACP supplementation significantly increased liver index, pancreas index, and bursa of Fabricius index (*P* < 0.05). The GSH-Px content of LPS-challenged broilers was lower than that of the control group (*P* < 0.001), but the content of MDA increased (*P* < 0.001). Feeding with 100 mg/kg ACP resulted in increased the activity of T-AOC, GSH-Px, and T-SOD, and decreased MDA content (*P* < 0.05). The activity of TNF-α, IL-1β, and IL-6 of the LPS group increased, but these indicators were decreased with supplemental 100 mg/kg ACP (*P* < 0.05). Dietary application of ACP up to 100 mg/kg down-regulated (*P* < 0.05) the expression of TLR4/NF-κB pathway in the liver induced by LPS. The results of 16S rRNA demonstrated that feeding with 100 mg/kg ACP can change the diversity and composition of the gut microbiota, and restrained the decline of beneficial cecal microbiota (typically *Lactobacillus, Faecalibacterium*, and *Christensenellaceae R-7* group) in the challenged LPS group (*P* < 0.05). Conclusively, feeding a diet with 100 mg/kg ACP may have beneficial effects on liver damage and the bacterial microbiota diversity and composition in the ceca of LPS-stressed slow-growing broiler breeds, probably because of its combined favorable effects on antioxidants and cytokines contents, and restoration the decline of beneficial cecal microbiota.

## Introduction

*Antrodia cinnamomea* is a medicinal distinctive mushroom, which is known in the china as “Niu-Chang-Chih” or the “Ruby of forest.” It is used in folk medicine as a cure for liver problems and a number of active substances can be found in *Antrodia cinnamomea* such as polysaccharides. Natural polysaccharides have various functions such as growth promotion, anti-inflammation, maintenance of intestinal mucosal integrity, regulation of intestinal flora (1–5). It has been reported that health benefits *of Antrodia cinnamomea* have antioxidants properties due to its polysaccharide, polyphenol and triterpenoid contents (6). *Antrodia cinnamomea* polysaccharide (ACP) has various bioactivities in mice (7, 8), piglets (9), and human (10, 11). Lee et al. (12) reported that ACP could promote Heme oxygenase−1 expression and antagonize the nuclear factor kappa B (NF-κB)-dominated inflammatory pathway. Therefore, the overall growth performance can be improved due to boost the immunity and antioxidant capacity in broiler chickens.

LPS is a specific component of the Gram-negative bacteria cell wall that can cause liver inflammation in chicken (13), mice (14) and piglets (10) with activated toll-like receptor 4 (TLR4), transmitting NF-κB into nucleus that can change the abundance of inflammatory genes. Liver diseases are reported to be associated with TLR4 medicated signals (15, 16), and it is also an important target of many therapeutic agents (13, 14, 17, 18). In addition, LPS was used to cause inflammatory model in chicken (13, 19, 20).

Gut microbiota is called “the new virtual metabolic organ” (21, 22), and the gut-liver axis has attracted greater attention (23, 24). As a center of body to connect external environment, gut not only provided the first defense protect intestine from hurt, but also supplied 70% blood of liver (24, 25). Previous studies suggested that TLRs and nod-like receptors mediated LPS, peptidoglycans and flagelin activated NF-κB to produce inflammatory cytokines and chemokines to cause liver steatosis, inflammation and fibrosis (24). Therefore, intestinal flora is an important participant in the regulation of liver diseases, and it is considered the core connotation of the enteric-liver axis theory.

Yellow feather chicken, as an important slow-growing broiler breeds. It is favored by consumers for its excellent meat quality and rich meat flavor. Nowadays, there are about 4 billion yellow-feathered chickens produced every year. Recently, it has been found that supplemental polysaccharides such as *Panax ginseng* (26) and *Quinoa* polysaccharide (27) can promote richness of species and improve the structure of gut microbiota community. The application of ACP in poultry production under LPS challenge on liver and microorganisms of slow-growing broiler breeds is not fully studied. Therefore, we hypothesized that ACP may have beneficial effects on LPS-induced liver damage and the variation of gut microbiota of slow-growing broiler breeds. The aim of this study was to investigate effects of ACP on liver antioxidant, anti-inflammatory capacity, and cecal flora structure of slow-growing broiler breeds challenged with LPS.

## Materials and methods

### Animals, experimental design and dietary treatments

The execution of this study followed the rules drawn by the Animal Care Committee belongs to the Institute of Animal Science, Guangdong Academy of Agricultural Sciences (GAASISA-2019-036).

A total of 750 Lingnan yellow-feathered female chicks (9-day-old) were purchased from Guangdong Wiz Agricultural Science and Technology Co. Ltd. (Guangzhou, China). On d 9, chicks were weighed (body weight = 137.69 ± 1.15 g) and divided into 30 floor pens (1.3 × 3.5 m) over a 21-day experimental period. Chicks were assigned to receive one of five treatments with six replicates of 25 chicks per replicate: a control diet, the chicks were fed a control diet and challenged with LPS. Dietary treatments 3 to 5 were the control diet supplemented with 100, 200, and 400 mg/kg ACP, respectively and challenged with LPS. On d 18 and 20 of age, 0.50 mL of normal saline was injected into peritoneum of birds in the control group, while 0.50 mL 500 μg/kg BW LPS was injected into peritoneum of birds in the challenged groups. The basic diet from 1 to 30 days (Table 1) was prepared according to Chinese Feeding Standard of Chicken (28).

**Table 1:** Composition and nutrient levels of basal diets (as-fed basis) %.

**Ingredients**	**%**
Corn	60.50
Soybean meal	31.5
Soybean oil	1.70
*L*-Lys · HCl	0.16
*DL*-Met	0.17
Limestone	1.22
CaHPO_4_	1.93
NaCl	0.30
Unite bran	1.52
Vitamin-mineral premix^a^	1.00
Total	100.00
Calculated nutrient composition	
ME, MJ/kg	12.12
CP	21.50
Lys	1.29
Met	0.52
Met+Cys	0.93
Thr	0.86
Trp	0.21
Ile	0.86
Ca	1.00
Total phosphorus	0.74
Non-phytate phosphorus	0.47

a The premix provided the following per kg of the diet: VA 15, 000 IU, VD_3_ 3, 300 IU, VE 10 IU, VK 0.50 mg, VB_1_ 1.8 mg, VB_2_ 3.6 mg, calcium pantothenate 10 mg, nicotinic acid 35 mg, VB_6_ 3.50 mg, biotin 0.15 mg, folic acid 0.55 mg, VB_12_ 0.01 mg, choline chloride 1,000 mg, Fe 80 mg, Cu 8 mg, Mn 80 mg, Zn 60 mg, I 0.35 mg, Se 0.15 mg.

ACP (*D-glucan*, 76.3%) was purchased from Taiwan Jia Shi Kai Biotechnology Co., Ltd. (Taibei, China). LPS generated by *E. coli* serotype O55: B5 (L4005) and was purchased from Sigma-Aldrich trading Co., Ltd. (Shanghai, China).

### Samples

On d 30, after deprived of feed overnight, all birds were weighed and 2 chickens (weight close to average body weight) were selected per pen then they were slaughtered after being anesthetized with Isoflurane (CPO406V2, Fresenius Kabi, Bad Homburg, Germany). The sample of liver and digesta of the cecum were transferred into sterile tubes. Then plunged into liquid nitrogen and stored at −80°C for further study. The liver, pancreas, spleen, thymus and bursa of Fabricius were dissected and weighed. Relative weight of organs was calculated by the organ weight /live weight × 100%.

### Biochemical indices in liver

The ice-cold physiologic saline (1:10, v/v) was used to homogenize samples of liver and then samples were centrifuged at 2,000 × g for 10 min. The activities of malondialdehyde (MDA), total superoxide dismutase (T-SOD), total antioxidant capacity (T-AOC) and glutathione peroxidase (GSH-Px) in liver were detected by colorimetric kits (Nanjing Jiancheng Institute of Bioengineering). Furthermore, the contents of interleukin 1β (IL-1β; Detection Range 1.00 ng/L~20.00 ng/L), interleukin 6 (IL-6; Detection Range 1.50 ng/L~30.00 ng/L) and tumor necrosis factor α (TNF-α, Detection Range 3.00 ng/L~80.00 ng/L) were detected by a spectrophotometer (Biomate 5, Thermo Electron Corporation, Rochester, NY, USA) with chicken Elisa kits (Beijing Equation Biotechnology co., Ltd, Beijing, China).

### The real-time quantitative PCR in liver

The method of targeted mRNA abundance detection has been described in Cui et al. (29). The gene primers were designed by Primer Premier 6.0 (Table 2), and generated by Sangon Biological Engineering Co., Ltd. (Shanghai, China). Total RNA from the liver of chickens were extracted by TRIzol Reagent (Thermo Fisher Scientific, Carlsbad, CA, USA) and checked for integrity by 1.5% agarose gel electrophoresis. Then, double-stranded DNA in RNA sample were eliminated by gDNA Remover (EZBioscience, Roseville, USA) and reacted at 25°C for 5 min. After that, 4 × RT Master Mix (EZBioscience, Roseville, USA) and primers were added and reacted at 42°C for 15 min to synthesized first strand cDNA. Relative mRNA abundance was calculated using the 2^−ΔΔCT^ with β*-actin* as the housekeeping gene.

**Table 2:** Primer sequences for quantitative real-time PCR.

**Genes^a^**	**Primer sequences (5'-3')**	**GenBank accession number**	**Annealing temperature /°C**	**PCR product size (bp)**
*TLR4*	F: AGTCTGAAATTGCTGAGCTCAAAT	NM_001030693.1	59	190
	R: GCGACGTTAAGCCATGGAAG			
*NF-κB*	F: CTACTGATTGCTGCTGGAGTTG	D13721.1	60	175
	R: CTGCTATGTGAAGAGGCGTTGT			
*COX2*	F: TGCAACGATATGGCTGAGAG	YP_009558655.1	58	233
	R: CTGCGATTCGGTTCTGGTAT			
*Nrf2*	F: ATCACCTCTTCTGCACCGAA	NM_205117.1	60	296
	R: GCTTTCTCCCGCTCTTTCTG			
*SOD1*	F: GGTGCTCACTTTAATCCTG	NM_205064.1	60	109
	R: CTACTTCTGCCACTCCTCC			
*BAX*	F: GTGATGGCATGGGACATAGCTC	CD214942.1	60	90
	R: TGGCGTAGACCTTGCGGATAA			
*β-actin*	F: GAGAAATTGTGCGTGACATCA	NM_205518	60	152
	R: CCTGAACCTCTCATTGCCA			

a *TLR4*, toll-like receptor (TLR)4; *NF-*κ*B*, nuclear factor kappa B; *COX2*, Cyclooxygenase 2; *Nrf2*, nuclear factor erythroid-2 related factor 2; *SOD1*, superoxide dismutase-1; *BAX*, B-cell lymphoma/leukemia-2-associated X protein; β*-actin*, beta actin.

### Determination of cecal microbiota

The QIAamp PowerFecal DNA Kit (Qiagen, Hilden, Germany) was used to extract total genomic DNA of cecal digesta. The Nanodrop 2000 spectrophotometer (Thermo Fisher Scientific, Wilmington, DE, United States) was used to measure DNA concentration. Then, the primer (Forward: 5'-CCTAYGGGRBGCASCAG-3' and Reverse: 5'- GGACTACNNGGGTATCTAAT-3') were used to amplify the bacterial 16S rRNA in the region of V3-V4. After that, the productions were purified by the AxyPrep DNA Gel Extraction Kit (Axygen Biosciences, Union City, CA, United States) and pooled in equimolar with paired-end sequenced (2 × 250) on an Illumina MiSeq platform (Shanghai BIOZERON Co., Ltd.) following standard protocols.

Based on a 97% sequence similarity cutoff by using the UPARSE software (https://drive5.com/uparse/, version 10), the operational taxonomic units (OTUs) were calculated and to identify unnormal gene sequences by UCHIME with the UCHIME software (http://drive5.com/usearch/manual/singletons.html). In each 16S rRNA gene sequence, the phylogenetic affiliation was analyzed by the Ribosomal Database Project (RDP) Classifier (https://rdp.cme.msu.edu/) against the Silva (SSU123) 16S rRNA database using a confidence threshold of 70% (30, 31). The rarefaction analysis, coverage abundance estimator, number of observed OTUs, diversity indices (Shannon and Simpson) and richness estimator (Chao 1 and ACE), which reflect bacterial diversity were calculated by mothur (version v.1.30.1) software. Based on the 97% sequence similarity, the phylum and genus levels were classified and the BLAST analysis of the OTUs against the SILVA database was used to determine Microbial species (30).

### Statistical analysis

Data were analyzed by the one-way analysis of variance (ANOVA) procedure and separated by Duncan's multiple range tests in SPSS 17.0 (SPSS Inc., Chicago, IL). Each replicate mean as the experimental unit for the growth performance data analysis, the other parameters were averaged per replicate. The control group and LPS-infected group were compared by *t*-tests. Orthogonal polynomial contrasts were also used to determine linear and quadratic responses of chickens to different levels of ACP supplementation. Data are presented as the means and pooled standard errors of the means (SEM). When *P*-value is less 0.05, the differences between treatments were considered statistically significant.

## Results

### Viscera indices

As exhibited in Table 3, the index of liver, pancreatic, and bursa of Fabricius were significantly elevated by 100~400 mg/kg ACP addition (*P* < 0.05). Supplemental ACP at 400 mg/kg increased the index of pancreas (*P* < 0.001) and spleen in linear trend (*P* = 0.022).

**Table 3:** Effects of *Antrodia cinnamomea* polysaccharide on organs indices of slow-growing broiler breeds challenged with LPS.

**Organs, mg/g**	**Treatments^a^**					**ACP^b^**			
	**Control**	**LPS**	**100 mg/kg ACP+LPS**	**200 mg/kg ACP+LPS**	**400 mg/kg ACP+LPS**	**SEM**	***P*** **-value**	**Linear**	**Quadratic**
Liver	28.46^a^	26.70^Bb^	30.30^a^	30.71^a^	31.99^a^	0.44	<0.001	<0.001	<0.001
Pancreas	3.25	2.92^c^	3.50^b^	3.83^a^^b^	3.92^a^	0.08	< 0.001	< 0.001	< 0.001
Spleen	1.73	1.64^b^	1.75^a^^b^	1.80^a^^b^	1.89^a^	0.04	0.153	0.022	0.073
Thymus	4.91	4.80	5.39	5.32	5.26	0.12	0.275	0.212	0.176
Bursa of Fabricius	3.05	2.94^b^	3.40^a^	3.47^a^	3.47^a^	0.08	0.038	0.015	0.016

Values are means of 6 replicates per treatment with 2 samples each. LPS, Lipopolysaccharide; ACP, Antrodia cinnamomea polysaccharide; SEM, standard error.

a Capital letters indicate statistically significant (*P* < 0.05) differences between control group and LPS group by Student's t-test; small letters indicate statistically significant (*P* < 0.05) differences of four groups (LPS, 100 mg/kg ACP+LPS, 200 mg/kg ACP+LPS, 400 mg/kg ACP+LPS). Mean values within a row with no common superscript differ significantly (*P* < 0.05).

b The *P*-value are representing the ANOVA analysis of four groups (LPS, 100 mg/kg ACP+LPS, 200 mg/kg ACP+LPS, 400 mg/kg ACP+LPS) and orthogonal polynomial contrasts are used to determine linear and quadratic responses of four groups.

### Antioxidant capacity and inflammatory factor of liver

As presented in Tables 4, 5, the activity of glutathione peroxidase (GSH-Px) in the liver of the broilers challenged with LPS was lower than that of the control group (*P* < 0.001), whereas the concentration of MDA was increased (*P* < 0.001). Supplemental 100~400 mg/kg ACP increased significantly the activities of T-AOC, GSH-Px and T-SOD and decreased the content of MDA in the liver (*P* < 0.05). The level of TNF-α, IL-1β and IL-6 were increased (*P* < 0.05) in the LPS group, but these levels were decreased by supplemental 100~400 mg/kg ACP (*P* < 0.05).

**Table 4:** Effects of *Antrodia cinnamomea* polysaccharide on liver antioxidant capacity of slow-growing broiler breeds challenged with LPS.

**Indices**	**Treatments^a^**					**ACP^b^**			
	**Control**	**LPS**	**100 mg/kg ACP+LPS**	**200 mg/kg ACP+LPS**	**400 mg/kg ACP+LPS**	**SEM**	***P*** **-value**	**Linear**	**Quadratic**
T-SOD, U/mg prot	464.54	427.59^b^	465.31^a^	483.77^a^	484.18^a^	6.98	0.005	0.001	0.002
GSH-Px, U/mg prot	37.53^A^	32.36^Bb^	40.05^a^	41.22^a^	43.84^a^	0.99	< 0.001	< 0.001	< 0.001
T-AOC, U/mg prot	0.91	0.81^b^	1.11^a^	1.11^a^	1.11^a^	0.03	< 0.001	0.001	< 0.001
MDA, nmol/mg prot	0.38^B^	0.78^Aa^	0.35^b^	0.33^b^	0.19^c^	0.04	< 0.001	< 0.001	< 0.001

Values are means of 6 replicates per treatment with 2 samples each. LPS, Lipopolysaccharide; ACP, Antrodia cinnamomea polysaccharide; GSH-Px, glutathione peroxidase; T-SOD, total superoxide dismutase; MDA, malondialdehyde; T-AOC, total antioxidant capacity; Prot, protein; SEM, standard error.

a Capital letters indicate statistically significant (*P* < 0.05) differences between control group and LPS group by Student's t-test; small letters indicate statistically significant (*P* < 0.05) differences of four groups (LPS, 100 mg/kg ACP+LPS, 200 mg/kg ACP+LPS, 400 mg/kg ACP+LPS). Mean values within a row with no common superscript differ significantly (*P* < 0.05).

b The *P*-value are representing the ANOVA analysis of four groups (LPS, 100 mg/kg ACP+LPS, 200 mg/kg ACP+LPS, 400 mg/kg ACP+LPS) and orthogonal polynomial contrasts are used to determine linear and quadratic responses of four groups.

**Table 5:** Effects of *Antrodia cinnamomea* polysaccharide on liver cytokines of slow-growing broiler breeds challenged with LPS.

**Indices, ng/g prot**	**Treatments^a^**					**ACP^b^**			
	**Control**	**LPS**	**100 mg/kg ACP+LPS**	**200 mg/kg ACP+LPS**	**400 mg/kg ACP+LPS**	**SEM**	***P*** **-value**	**Linear**	**Quadratic**
TNF-α	4.95^B^	6.12^Aa^	4.24^b^	4.22^b^	3.94^b^	0.20	< 0.001	< 0.001	< 0.001
IL-1β	1.57^B^	2.04^Aa^	1.33^b^	1.32^b^	1.11^b^	0.08	< 0.001	< 0.001	< 0.001
IL-6	2.47^B^	2.83^Aa^	2.01^bc^	2.19^b^	1.77^c^	0.09	< 0.001	< 0.001	< 0.001

Values are means of 6 replicates per treatment with 2 samples each. LPS, Lipopolysaccharide; ACP, Antrodia cinnamomea polysaccharide; TNF-α, tumor necrosis factor α; IL-1β, interleukin 1β; IL-6, interleukin 6. prot, protein; SEM, standard error.

a Capital letters indicate statistically significant (*P* < 0.05) differences between control group and LPS group by Student's t-test; small letters indicate statistically significant (*P* < 0.05) differences of four groups (LPS, 100 mg/kg ACP+LPS, 200 mg/kg ACP+LPS, 400 mg/kg ACP+LPS). Mean values within a row with no common superscript differ significantly (*P* < 0.05).

b The *P*-value are representing the ANOVA analysis of four groups (LPS, 100 mg/kg ACP+LPS, 200 mg/kg ACP+LPS, 400 mg/kg ACP+LPS) and orthogonal polynomial contrasts are used to determine linear and quadratic responses of four groups.

### The mRNA expression abundance of antioxidant and anti-damage genes of liver

**Figure 1:**
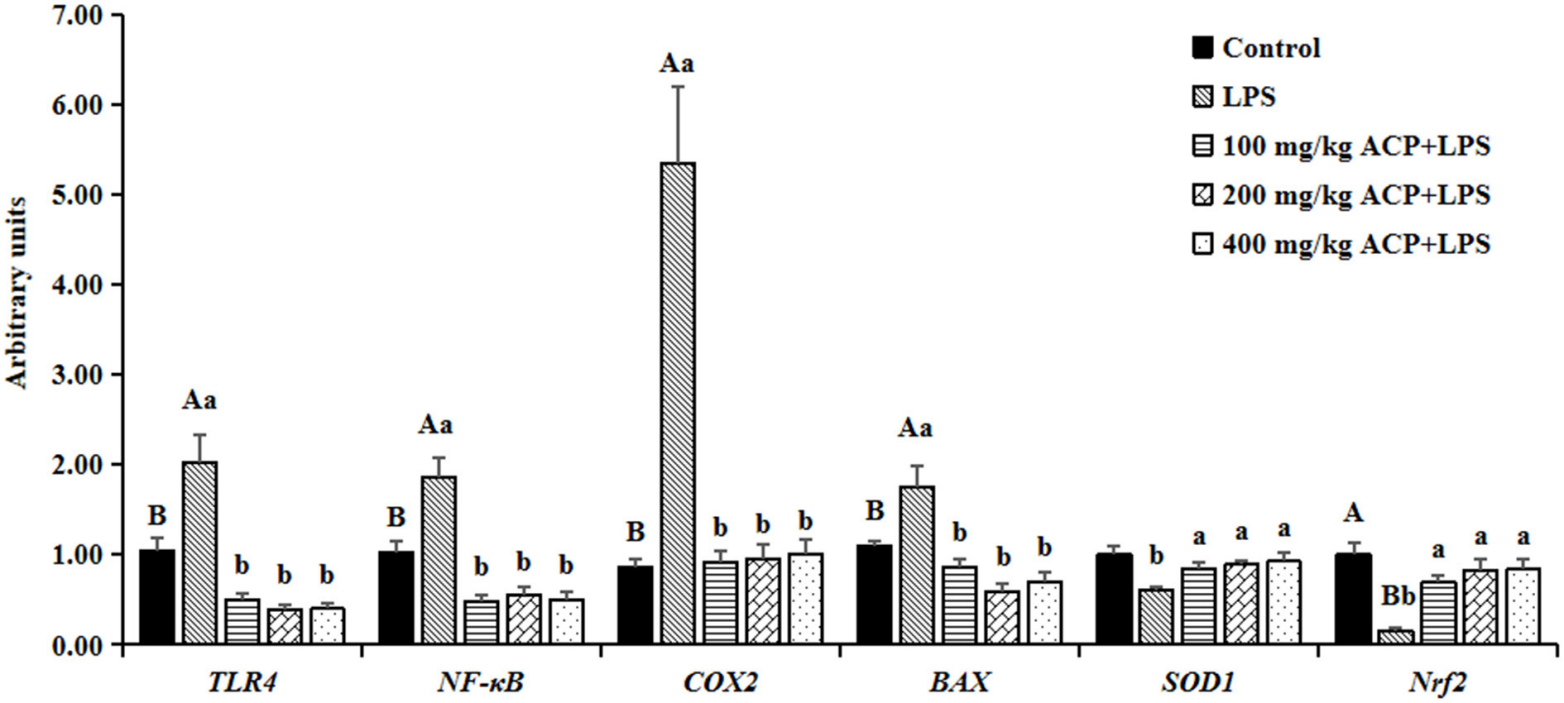
Effects of *Antrodia cinnamomea* polysaccharide on mRNA expression abundance of antioxidant and anti-damage genes in liver of slow-growing broiler breeds challenged with LPS^*a*^. Values are means of 6 replicates per treatment with 2 samples each. LPS, Lipopolysaccharide; ACP, *Antrodia cinnamomea* polysaccharide; *TLR4*, toll-like receptor (*TLR*)*4*; *NF-*κ*B*, nuclear factor kappa B; *COX*2, Cyclooxygenase 2; *Nrf2*, nuclear factor erythroid-2 related factor 2; *SOD1*, superoxide dismutase-1; *BAX*, B-cell lymphoma/leukemia-2-associated X protein; Control, basal diet group; LPS, basal diet+LPS stress group. 100 mg/kg ACP+LPS, basal diet+LPS stress+100 mg/kg ACP group. 200 mg/kg ACP+LPS, basal diet+LPS stress+200 mg/kg ACP group. 400 mg/kg ACP+LPS, basal diet+LPS stress+400 mg/kg ACP group. ^*a*^Data are expressed as means±SEM. SEM, standard error. Capital letters indicate statistically significant (*P* < 0.05) differences between control group and LPS group by Student's *t*-test; small letters indicate statistically significant (*P* < 0.05) differences of four groups (LPS, 100 mg/kg ACP+LPS, 200 mg/kg ACP+LPS, 400 mg/kg ACP+LPS). Bars with no common superscript differ significantly (*P* < 0.05).

As shown in Figure 1, LPS notably elevated the mRNA abundance of *NF-*κ*B, TLR4, COX2* and *BAX* (*P* < 0.05), while the *Nrf2* abundance was significantly decreased (*P* < 0.05) in the liver of slow-growing broiler breeds compared with the control group. The supplemental ACP inhibited the LPS-induced *TLR4, NF-*κ*B, COX2* and *BAX* mRNA expression in a dose-independent model (*P* < 0.05). Meanwhile, the mRNA abundance of *Nrf2* was drastically elevated and *SOD1* was slight up-regulated in the different ACP pretreatment groups (*P* < 0.05). However, there was no significantly changes on the mRNA abundance of antioxidant and anti-damage genes in the liver between the different doses of ACP group (*P* > 0.05).

### Cecal microflora

A total of 2,285,846 16S rRNA effective sequences in V3-V4 region from the 48 samples, and 47,622 sequences per sample were used for further study. The average read length was 414 bp. In Table 6, the number of OTUs, sample richness and diversity are displayed. Coverage of each group was higher than 0.99, suggested that the sequencing results had a great Coverage rate and could reflect the distribution of cecal microorganisms in slow-growing broiler breeds. The Shannon index of 400 mg/kg ACP group was significantly higher than that in the LPS group. Compared with the LPS group, Simpson index of the ACP groups were all significantly lower. Additionally, compared to the LPS group, there was a significant linear effect in the Shannon of chickens that received the ACP-supplemented diet without dose dependence (*P* < 0.05). Moreover, there was a obvious linear and quadratic effect on the Simpson of chickens that fed the ACP diet in dose-independent compared to LPS stress group (*P* < 0.05).

**Table 6:** Effects of *Antrodia cinnamomea* polysaccharide on Alpha diversity analysis of the microbiota from cecum of yellow-feathered chicken based on 97% sequence similarity.

**Indices**	**Treatments^a^**					**ACP^b^**			
	**Control**	**LPS**	**100 mg/kg ACP+LPS**	**200 mg/kg ACP+LPS**	**400 mg/kg ACP+LPS**	**SEM**	***P*** **-value**	**Linear**	**Quadratic**
OTU numbers	1,228.83	1,137.83	1,258.33	1,189.67	1,260.33	31.82	0.485	0.304	0.555
Coverage, %	99.14	99.23	99.31	99.33	99.24	0.04	0.734	0.861	0.528
**Richness**									
Chao1	1,617.65	1,469.03	1,560.71	1,529.17	1,606.31	33.98	0.568	0.218	0.474
ACE	1,613.46	1,462.86	1,570.71	1,509.72	1,606.82	34.48	0.487	0.237	0.504
**Diversity indices**									
Shannon	4.34	4.12^b^	4.54^a^^b^	4.65^a^^b^	4.73^a^	0.10	0.142	0.027	0.063
Simpson	0.07	0.10^a^	0.05^b^	0.04^b^	0.04^b^	0.01	0.006	0.003	0.002

Values are means of 6 replicates per treatment. LPS, Lipopolysaccharide; ACP, Antrodia cinnamomea polysaccharide; OUT, operational taxonomic units; ACE, abundance-based coverage estimator; SEM, standard error.

a Capital letters indicate statistically significant (*P* < 0.05) differences between control group and LPS group by Student's t-test; small letters indicate statistically significant (*P* < 0.05) differences of four groups (LPS, 100 mg/kg ACP+LPS, 200 mg/kg ACP+LPS, 400 mg/kg ACP+LPS). Mean values within a row with no common superscript differ significantly (*P* < 0.05).

b The *P*-value are representing the ANOVA analysis of four groups (LPS, 100 mg/kg ACP+LPS, 200 mg/kg ACP+LPS, 400 mg/kg ACP+LPS) and orthogonal polynomial contrasts are used to determine linear and quadratic responses of four groups.

**Figure 2:**
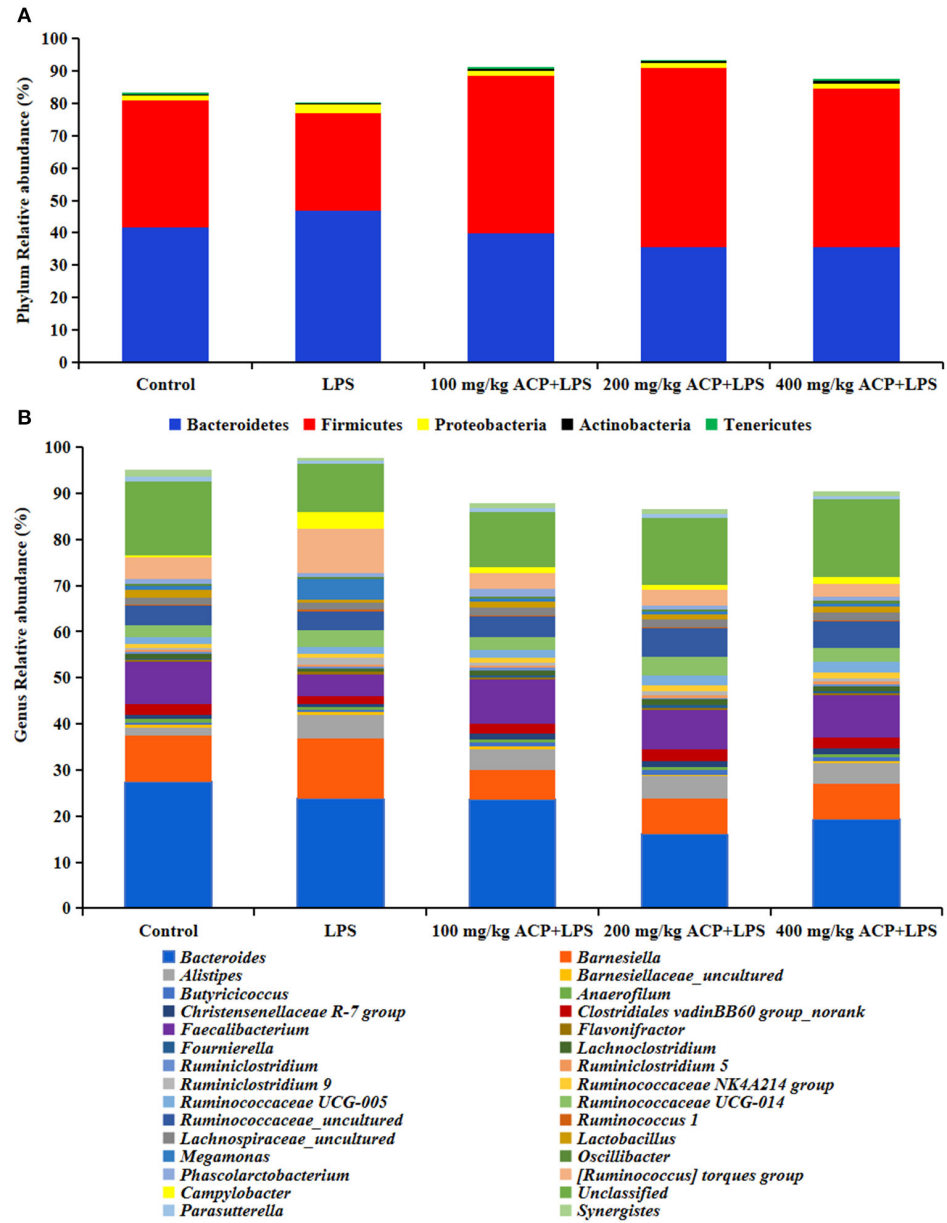
Effects of *Antrodia cinnamomea* polysaccharide on microbiome composition in the cecum of slow-growing broilers challenged with LPS. **(A)** Composition and distribution of the microbiota at the phylum level. **(B)** Composition and distribution of the microbiota at the genus level. Values are means of 6 replicates per treatment. LPS, Lipopolysaccharide; ACP, *Antrodia cinnamomea* polysaccharide; Control, basal diet group; LPS, basal diet+LPS stress group; 100 mg/kg ACP+LPS, basal diet+LPS stress+100 mg/kg ACP group. 200 mg/kg ACP+LPS, basal diet+LPS stress+200 mg/kg ACP group. 400 mg/kg ACP+LPS, basal diet+LPS stress+400 mg/kg ACP group.

As shown in Figure 2 and Table 7, compared with the control group, LPS reduced Firmicutes abundance (*P* > 0.05) and increased Proteobacteria abundance (*P* < 0.05). However, compared to the LPS group, significant increases of Firmicutes abundance and significant decreases of Proteobacteria abundance of slow-growing broiler breeds were observed in the different ACP groups (*P* < 0.05). Intriguingly, the Actinobacteria and Tenericutes abundance were significantly elevated in the 400 mg/kg ACP group compared to the LPS group. There was a significant linear and quadratic effect in the phylum level (except Bacteroidetes) of chickens fed with ACP diet compared with the LPS stress group (*P* < 0.05).

**Table 7:** Effects of *Antrodia cinnamomea* polysaccharide on microbiome composition in the cecum of slow-growing broiler breeds challenged with LPS.

**Parameter**	**Treatments^a^**					**ACP^b^**			
	**Control**	**LPS**	**100 mg/kg ACP+LPS**	**200 mg/kg ACP+LPS**	**400 mg/kg ACP+LPS**	**SEM**	***P*** **-value**	**Linear**	**Quadratic**
**Phylum relative abundance (%)**									
Bacteroidetes	41.78	46.69	39.95	35.42	35.53	2.20	0.225	0.047	0.106
Firmicutes	39.14	30.29^b^	48.41^a^	55.61^a^	49.00^a^	2.52	<0.001	0.002	<0.001
Proteobacteria	1.34^B^	2.56^Aa^	1.73^b^	1.41^b^	1.64^b^	0.14	0.003	0.003	0.001
Actinobacteria	0.52	0.42^b^	0.46^b^	0.53^a^^b^	0.77^a^	0.05	0.042	0.007	0.015
Tenericutes	0.14	0.15^b^	0.23^a^^b^	0.19^a^^b^	0.28^a^	0.02	0.032	0.012	0.048
**Genus relative abundance (%)**									
*Alistipes*	1.79^B^	5.08^A^	4.62	4.92	4.60	0.24	0.896	0.644	0.895
*Christensenellaceae R-7 group*	0.74	0.60^b^	1.18^a^	1.25^a^	1.17^a^	0.09	0.013	0.019	0.004
*Clostridiales vadinBB60 group_norank*	2.43	1.54	2.30	2.49	2.27	0.16	0.228	0.164	0.105
*Faecalibacterium*	9.21	4.88^b^	9.46^a^	8.65^a^	9.20^a^	0.54	0.003	0.013	0.006
*Ruminiclostridium 9*	0.43^B^	1.55^Aa^	0.80^b^	0.80^b^	0.82^b^	0.10	0.004	0.011	0.002
*Ruminococcus 1*	0.12	0.38^a^	0.22^b^	0.21^b^	0.20^b^	0.03	0.034	0.017	0.016
*Lactobacillus*	1.86^A^	0.62^Bc^	1.14^a^^b^	1.09^b^	1.46^a^	0.09	0.002	0.001	0.003
*Megamonas*	0.67^B^	4.39^Aa^	0.64^b^	0.49^b^	0.54^b^	0.51	0.035	0.023	0.016
*[Ruminococcus] torques group*	4.61^B^	9.76^Aa^	3.47^b^	3.32^b^	2.91^b^	0.82	< 0.001	0.001	0.000
*Campylobacter*	0.41^B^	3.53^Aa^	1.32^b^	1.16^b^	1.37^b^	0.29	<0.001	0.005	0.000
*Synergistes*	1.49^A^	0.57^Bb^	1.07^a^	1.14^a^	0.97^a^^b^	0.08	0.058	0.118	0.020
*Unclassified*	16.12	10.49^b^	11.96^b^	14.50^a^^b^	16.83^a^	0.88	0.026	0.001	0.008

Values are means of 6 replicates per treatment. LPS, Lipopolysaccharide; ACP, Antrodia cinnamomea polysaccharide; SEM, standard error.

a Capital letters indicate statistically significant (*P* < 0.05) differences between control group and LPS group by Student's t-test; small letters indicate statistically significant (*P* < 0.05) differences of four groups (LPS, 100 mg/kg ACP+LPS, 200 mg/kg ACP+LPS, 400 mg/kg ACP+LPS). Mean values within a row with no common superscript differ significantly (*P* < 0.05).

b The *P*-value are representing the ANOVA analysis of four groups (LPS, 100 mg/kg ACP+LPS, 200 mg/kg ACP+LPS, 400 mg/kg ACP+LPS) and orthogonal polynomial contrasts are used to determine linear and quadratic responses of four groups.

At the genus level, LPS increased the relative abundance of *Alistipes, Ruminiclostridium 9, Megamonas, [Ruminococcus] torques* group and *Campylobacter* (*P* < 0.05), and decreased the relative abundance of *Lactobacillus* and *Synergistes* (*P* < 0.05). Interestingly, the changes of *Christensenellaceae R-7, Faecalibacterium, Ruminiclostridium 9, Ruminococcus 1, Lactobacillus, Megamonas, [Ruminococcus] torques* group, *Campylobacter* and *Synergistes* were reversed by the 100~400 mg/kg ACP-supplemented diet (*P* < 0.05).

## Discussion

It is well known that that LPS induces stress response in broilers that leads to redistribute nutrients to synthesize antibodies, which reduces nutrients required for broiler growth (32, 33). LPS can damage intestinal tissue structure and intestinal mucosal barrier, increase the expression of inflammatory factors, and trigger the inflammatory response of broilers (20, 34). On the other hand, it has been found that polysaccharide can improve gut functions. For example, dietary *Algae-Derived* polysaccharide supplementation had an ameliorative effect on heat stress-induced impairment of tight junctions, antioxidant capacity and the immune response of the duodenum in broilers (35). In addition, *Ganoderma lucidum* polysaccharides and *Agaricus blazei* polysaccharides can attenuate the cadmium-induced oxidative damage by decreasing the GSH-Px and SOD activity, and decrease the level of MDA (36). Furthermore, the dietary *Algae-Derived* polysaccharides (37) and *Lycium barbarum* polysaccharides (38) supplementation can elevate the SOD activities and reduce the MDA levels in liver of broilers. In current study, the activity of anti-inflammatory cytokines and antioxidants in the liver were improved by supplemental ACP, and the index of liver and bursa of Fabricius were increased by ACP addition.

Hepatic inflammation and oxidative stress induced by LPS are closely linked with TLR4/NF-κB pathway (39–41). LPS molecule was recognized by TLR4 at the Gram-negative bacteria outer membrane and activated strong immune response via NF-κB (14, 42). It was suggested that oxidative stress activated NF-κB by altering the expression of *COX2, IL-1*β, *IL-6* and *TNF-*α to induce inflammation (18, 43, 44). Consistently, these results displayed that LPS decreased the level of GSH-Px and increased the content of MDA in liver. Moreover, these data were in accordance with previous publications of Han et al. (39) and Mei et al. (45) as they found that LPS increased mRNA level of *TLR4, TNF-*α, *NF-*κ*B, IL-1*β, *COX-2, IL-6* and *BAX*, and decreased the expression of *Nrf2* in the liver of broilers. In the present study, it has been showed that LPS caused negative affect on liver health, which was consistent with the finding by Cheng et al. (13) on Beijing white chickens.

Dietary supplemented with *Artemisia ordosica* polysaccharide obviously alleviated LPS-caused oxidative stress through TLR4/NF-κB pathway (41). Recent discoveries suggested that *Ganoderma lucidum* polysaccharides and *Agaricus blazei* polysaccharides inhibited the TLR4 signaling pathway and weakened the damage caused by cadmium in chickens (36). Dietary *Algae-Derived* polysaccharide ameliorated the impairment of histology, cell apoptosis and immune balance in bursa of Fabricius of heat stressed broilers, which is involved in modulation of NF-κB signaling pathway (5). Interestingly, liver damage was significantly alleviated by ACP. All data showed that ACP may have positive effects on LPS-caused liver inflammation by rescue the oxidative stress *via* activation of the TLR4/NF-κB pathway in chicken.

Liver diseases are linked to intestinal dysbiosis. Several studies claimed that gut microbiota affected liver pathophysiology directly or indirectly through a series of signaling pathways (23–25). Polysaccharides can regulate the composition of intestinal flora and have an effect on the level of the host intestinal flora (46). As an important link for the interaction between the body and polysaccharides, the study of intestinal flora should not be ignored. After glycolysis by intestinal microorganisms, polysaccharides can promote the growth of probiotics and intestinal biodiversity (47, 48). In the current study, dietary supplemented with 100 mg/kg ACP increased species richness and diversity indices, which were decreased by LPS in the present study. This was reflected by the Shannon index and Simpson index with statistical differences.

It has been reported that *Panax ginseng* polysaccharides changed the diversity and composition of the gut microbiota with antibiotic-associated diarrhea in mice (26). *Panax ginseng* polysaccharides can increase phylum Firmicutes abundance and decrease phyla Bacteroidetes, Proteobacteria and Actinobacteria abundance (26). *Quinoa* polysaccharide enhanced species richness by regulating the community structure of gut microbiota, reducing the ratio of Firmicutes to Bacteroides and Proteobacteria abundance in rats (27). This study showed that Firmicutes and Bacteroidetes were the greatest predominant phylum in caeca of slow-growing broilers, which was inconsistent with previous reports (49, 50). Meanwhile, our results revealed that ACP was inhibited the decline of *Faecalibacterium* (typically Firmicutes) and the rise of Proteobacteria in LPS-induced group. Accordingly, Lee et al. (12) reported that a linear decrease was detected in the cecum coliform (Proteobacteria) count by ACP supplementation in comparison with the control group. Notably, ACP restrained beneficial cecal microbiota reduction (typically *Lactobacillus* and *Christensenellaceae R-7* group) in LPS-induced chickens. Consistently, the lactic acid bacteria showed an increased tendency by ACP supplementation (12). Moreover, Zhang et al. (48) has concluded that plant bioactive substances produced metabolites by gut microbiota in the distal gastrointestinal. The results of this study also support the function of ACP in the rescue of a normal microbial environment exerting a prebiotic promotion of beneficial bacteria.

The liver is an important metabolic organ and it is a multi-purpose organ including, clearing excess-production of ROS to maintain oxidative balance by decreasing MDA observed after LPS-challenge herein. In addition, it has been reported that polysaccharide can reach the liver *via* the portal vein after absorption along the gastrointestinal tract (51). Therefore, the potential beneficial effect of ACP as an antioxidant would be expected primarily in liver. On the other side, gut microbiota dysbiosis is associated with oxidative stress (52). In addition, the relationship between oxidative stress and gut microbiota has been illustrated (53). It has been found that oxidative stress alters the oxygen gradient and shifts the equilibrium of microbiota from anaerobic to facultative anaerobic groups (54). Therefore, the gut health (microbiome) can interact tightly with the liver that called a “gut-liver axis”. This axis offers liver specific interacts with a substantial amount of microbiota derived signals (24). Accordingly, it can be inferred that feeding a diet with ACP may help to alleviate the oxidative stress in the liver and regulate the microbiota composition in ceca after LPS-challenge in the present study.

## Conclusions

Feeding a diet with 100 mg/kg ACP may have beneficial effects on liver damage and the bacterial microbiota diversity and composition in the ceca of LPS-stressed slow-growing broiler breeds, probably because of its combined favorable effects on antioxidants and cytokines contents, and inhibition of the expression of TLR4/NF-κB signaling pathway in the liver, and restoration the decline of beneficial cecal microbiota (typically *Lactobacillus, Faecalibacterium*, and *Christensenellaceae* R-7 group).

## Data availability statement

The datasets presented in this study can be found in online repositories. The names of the repository/repositories and accession number(s) can be found in the article/supplementary materials.

## Ethics statement

The animal study was reviewed and approved by Animal Care Committee of the Institute of Animal Science, Guangdong Academy of Agricultural Sciences.

## Author contributions

JY, CZ, and MA performed experiments, analyzed data, and wrote the manuscript. QF, XL, and YW performed experiments. RA, AA, and SJ supervised the project, developed the study concept, and wrote and edited the manuscript. All authors read and approved the final manuscript.

## Funding

This work was financially supported by the Key Realm R&D Program of Guangdong Province (2020B0202090004), China Agriculture Research System of MOF and MARA (CARS-41), National Key R&D Project (2021YFD1300404), Natural Science Foundation from Guangdong Province (2022A1515012069, 2021A1515012412, and 2021A1515010830), the Science and Technology Plan Project of Guangzhou (202206010168), the Science and Technology Program of Guangdong Academy of Agricultural Sciences (202106TD, R2019PY-QF008), Introduction of Talents Program from Guangdong Academy of Agricultural Sciences (R2021YJ-YB3012), Guiding Agreement of Young Scholar from Guangdong Academy of Agricultural Sciences (R2021QD-024) P. R. China, and Research Supporting Project (RSP-2022R439), King Saud University, Riyadh, Saudi Arabia.

## Conflict of interest

The authors declare that the research was conducted in the absence of any commercial or financial relationships that could be construed as a potential conflict of interest.

## Publisher's note

All claims expressed in this article are solely those of the authors and do not necessarily represent those of their affiliated organizations, or those of the publisher, the editors and the reviewers. Any product that may be evaluated in this article, or claim that may be made by its manufacturer, is not guaranteed or endorsed by the publisher.

## Abbreviations

LPS: Lipopolysaccharide
ACP: *Antrodia cinnamomea* polysaccharide
GSH-Px: glutathione peroxidase
T-AOC: total antioxidant capacity
T-SOD: total superoxide dismutase
MDA: malondialdehyde
TNF-α: tumor necrosis factor α
IL-1β: interleukin 1β
TLR4: toll-like receptor (TLR)4
IL-6: interleukin 6
NF-κB: nuclear factor kappa B
COX2: Cyclooxygenase 2
Nrf2: nuclear factor erythroid-2 related factor 2
SOD1: superoxide dismutase-1
BAX: B-cell lymphoma/leukemia-2-associated X protein.
